# Effects of time and temperature during melanging on the volatile profile of dark chocolate

**DOI:** 10.1038/s41598-020-71822-0

**Published:** 2020-09-10

**Authors:** Caitlin Clark, Harmonie M. Bettenhausen, Adam L. Heuberger, Jeffrey Miller, Linxing Yao, Martha Stone

**Affiliations:** 1 Department of Food Science and Human Nutrition, Colorado State University, Fort Collins,, CO USA; 2 Nuance Chocolate, Fort Collins, CO USA; 3 Department of Horticulture and Landscape Architecture, Colorado State University, Fort Collins, CO USA; 4 Proteomics and Metabolomics Facility, Colorado State University, Fort Collins, CO USA

**Keywords:** Mass spectrometry, Cheminformatics, Metabolomics, Small molecules

## Abstract

Chocolate made from small-batch production is known for distinct sensory properties that differentiate its products from large-scale production. Specifically, small-batch processing includes a melanging step, a chocolate refining (a process involving time and temperature to refine texture and flavor) process that occurs in a stone wet-grinder. Chocolatiers understand that melanging is essential to flavor and overall quality, however the influence of melanging on the aroma chemistry of the finished chocolate is anecdotal and largely uncharacterized. Here, we evaluated the effects of time and temperature of melanging on the volatile chemistry of the finished chocolate. Specifically, chocolate aroma was profiled using HS/SPME–GC–MS for three different time and temperature combinations. A total of 88 compounds were annotated by mass spectrometry and included a diverse set of chemical classes such as pyrazines, aldehydes, terpenes, alcohols, esters, and ketones. Analysis of variance (ANOVA), principal component analysis (PCA), and partial least squares analysis (PLS) revealed that the overall aroma profile was influenced by the type of melanging, and time had a greater effect than temperature. Example compounds affected by time include 2-methylpropanal, dimethyl sulfide, and benzaldehyde. Particle size was also measured for each sample. Majority particle size was found to be below 25 microns generally at all time points beyond 8 h. Analysis showed significant *p*-values for the temperature variable for several compounds, but significant *p*-values for the time variable were apparent for a greater number of compounds. For compounds which showed dependency on both time and temperature, the *p*-value for the time variable was much smaller in most cases. Both PCA and OPLS analyses suggested the same trends. These data support that time is a critical factor in determining the aroma chemistry of chocolate and affects a diverse set of known flavor active compounds.

## Introduction

Chocolate is a confection made from the seeds of *Theobroma cacao,* one of 22 species of the genus *Theobroma*^1^. Several of these species are used in the preparation of food and beverages, but *T. cacao* is most commonly used, and it results—with some minimal contribution in certain locals by *T. bicolor* and *T. grandiflorum*—in the foods and drinks collectively known as chocolate^2^. Modern-day chocolate is prepared in an elaborate series of fermentation, drying, grinding, roasting, and fat crystallization processes.

Small-batch chocolate processing follows roasting, winnowing, and de-husking steps by refining chocolate with sugar and occasionally other ingredients, usually in a type of stone grinder called a melanger^3^. This process was laid out for large-scale producers in *State-of-the-Art Chocolate Manufacture: A Review*^4^ which offers an excellent review and discussion of large-scale industrial chocolate processing. One essential aspect of this refining step, regardless of how it takes place, is achieving the appropriate particle size. Above a particle size of 30 microns, the tongue is still able to distinguish individual textures^5^. However, below 4 microns, excessive surface area becomes available for the fats to coat, and the chocolate may be perceived as slimy and too viscous^6^ with a high Casson yield value due to increased particle interactions^5^. For this reason, most producers aim for a particle size between 15–25 microns^7^. When the chocolate is smooth, it undergoes a series of controlled heating and cooling steps to crystalize the fats into the most desirable structure in a process known as “tempering”^8^.

Melangers from Cocoatown and other producers such as Cacao Cucina are now the most common flavor and particle size refining tool used by small-batch chocolate makers because of their scalability and low upfront capital investment^9^. Despite this, there is little academic literature on the use of melangers for chocolate-making, except that literature which tests their utility as conch-like substitute^3,10,11^, which is not reflective of how they are typically used by chocolate-makers. Melanging can be distinguished from conching in that, in a real-world application by small-batch chocolate-makers, melangers are typically used at ambient temperature (whereas conches are generally heated to up to 80 °C^12^) and melangers include a particle-size refining step^13,14^ (whereas traditional conching requires that the chocolate mass be refined first^15^).

Therefore, the goal of this study was to determine the effects of time and temperature on melanging outcome by measuring volatile profiles before, after, and at two stages during melanging under three different temperature treatments. Particle size was also determined at teach temperature and time point. By comparing these results to what is already known about conching, a further objective was to determine if melanging is an acceptable alternative to conching and to probe whether pre-existing data on conching might be extrapolated to melanging systems.

## Materials and methods

### Chocolate production

Raw materials and a bench-scale melanger were provided by Nuance Chocolate (Fort Collins, Colorado, USA). Raw cacao beans were sourced from Ghana and stored at 24 °C until used in this study. For chocolate production, 478 g of cacao was sorted to remove any stones, insects, or other non-cacao materials that could interfere with processing. Beans were roasted for 17 min at 163 °C starting from guidelines suggested for larger volumes by Gibson and Newson (2018)^8^ followed by trial roasts to adjust for smaller volumes. Roasted beans were then removed from the oven and cooled to room temperature. Next, the roasted cacao beans were passed through a juicer (Champion, Lodi, CA, USA) connected to a Hoover L2310 vacuum (Hoover, Glenwillow, OH, USA) with a 10 Amp motor, for a process known as cracking and winnowing, which shatters the beans and removes the thin outer husk, resulting in a product known as cacao nibs. A total of 350 g of nibs were placed into a Spectra 11 bench-size stone melanger (Spectra, Coimbatore, India), followed immediately by 25 g of liquified cocoa butter and 125 g of superfine cane sugar. When all ingredients had combined into a visibly homogenous slurry (within five minutes of being mixed), a 0-h time-point sample was collected from each batch. The melanger was then placed into a Caron 7000-25 series incubation chamber (Caron, Mariette, OH, USA) to control ambient temperature for each of the three temperature treatments (16 °C, 24 °C, and 38 °C). Further samples were taken at 8-h, 16-h, and 24-h time points. Each sample consisted of 2 g of chocolate mass piped into a cylindrical glass SPME vial using a 1 ml syringe, then further sealed inside a plastic container and incubated at 4 °C until chemical analysis.

### Particle size

Particle size measurements were measured using a stainless steel 0–50 micron grindometer (Boshi Electronic Instruments, Baiyun, Guangzhou, China). Results were expressed as a range of majority particle size, based on visual analysis of the grindometer. Owen^16^ explains that a particle size below 30 microns is desirable in chocolate for a smooth and finished texture because the tongue is unable to determine specific textures at this small diameter. Other authors concur, some claiming that 20 microns is the ideal particle size for finished chocolate^17,18^. Tan^7^ states explicitly that a range of 15 to 25 microns is most desirable. By this definition, these results indicate that the chocolate samples could have been classified as “finished” with regard to particle size.

### Aroma chemistry detection and data processing

Aroma compounds from the finished chocolate were detected using headspace solid phase microextraction gas chromatography mass spectrometry (HS/SPME–GC–MS). Methods were derived from Rodriguez-Campos et al.^19^ with minor modifications. Workflow was also informed by methods used by Torres-Moreno et al.^20^ as well as Quin et al.^21^ and Albak and Tekin^12^. Briefly, 2 g of each sample, while still in a liquid state, was pipetted into the 20-mL SPME vial using a 1 mL syringe, then sealed in the flask and exposed to a divinylbenzene/carboxen/polydimethylsiloxane (DVB/CAR/PDMS) 50/30 m SPME fiber for 10 min at 60 °C to equilibrate, followed by a 20 min extraction with agitation at the same temperature. Trace1310 GC (Thermo) was coupled to a Thermo ISQ-LT MS. Volatile compounds were desorbed at 250 °C for 5 min into a DB-WAXUI Ultra Inert column (30 m × 0.25 mm × 0.25 µm, www.agilent.com). The inlet was operated in splitless mode. A constant flow rate of the carrier gas (He) was controlled at 1.2 mL/min. The GC ramping included: 40 °C for 3 min, then increase of 10 °C/min to 230 °C, then to 250 °C at rate of 20 °C/min and held for 1 min. The total run time per sample was approximately 60 min. The MS was operated at 70 eV in electron impact mode, and the transfer line and ion source temperatures were both set at 250 °C. Detection was performed in full scan mode from 40–400 amu.

### Detection of volatile metabolites in chocolate and metabolomics data processing

Volatile metabolites in chocolate were detected using a non-targeted metabolomics approach via headspace solid-phase microextraction gas chromatography–mass spectrometry (HS/SPME–GC–MS). The workflow was performed as previously reported in Bettenhausen et al.^22^, and the HS/SPME–GC–MS settings are described in the previous section. Mass spectra from the HS/SPME–GC–MS analysis were converted to the .cdf file format, and peak detection, grouping, retention time alignment, and peak filling was performed using XCMS algorithms in the R statistical environment (v 3.5.1)^23,24^. The molecular features were deconvoluted into spectral clusters using the RAMclustR package in R^25^. The outputs of this data processing workflow resulted in a data set consisting of metabolites defined by (i) mass spectra, (ii) retention time, and (iii) relative abundance and normalized to the total ion current as reported previously^25^. Metabolites were identified to MSI Confidence Level 2 by spectral matching in NIST MS Search software to an in-house database of ~ 1,500 compounds and to external and theoretical databases including NIST v14 (https://www.nist.gov) and the Human Metabolome Database^26^.

### Experimental design/statistics

Volatile metabolite abundances were compared using two-way analysis of variance (ANOVA) via the *aov* function in the R statistical environment v. 3.5.1^27^, and false discovery rate adjustment was performed on the ANOVA p-values using the Benjamini–Hochberg algorithm^28^ Principal Components Analysis (PCA), and Orthogonal Partial Least Squares Analysis (OPLS) were conducted on 88 annotated metabolites with SIMCA software v. 15 (Sartorius Stedim Biotech, Umea, Sweden). Scores and loadings values from OPLS models were conducted in SIMCA software^29^ on data for time and temperature (y) and annotated, unit variance scaled volatile metabolites (x). Predictive power (Q2) was determined via cross-validation, by which the data was divided into seven parts and 1/7th of the data were removed, and the model was built on the remaining 6/7th of data, and the removed 1/7th of data are predicted from the model.

Prior to heat-mapping, volatile metabolite data were normalized within each time and temperature point via z-transformation (normalized peak area – mean peak area/standard deviation of total peak area of each metabolite). The resulting z-scores were converted into colors and grouped using hierarchical clustering on the Spearman’s rank correlation (r_s_) between metabolite and sensory trait values^30^. Correlation plots were generated in R using the gplots, ggplots2, Reshape2, and stats packages via the *heatmap.2*, *melt,* and *hclust* functions^31^.

## Results

### Melanging time and temperature and chocolate particle size

In this study, all samples tested other than those at the zero time points showed most particle sizes were below 30 microns. As expected, particle size continued to drop as melanger time was increased (Table 1). The particle size data support that the chocolate used in this study was indeed “finished”, at any point beyond 8 h, as all samples at 8 h and beyond met the criteria of having a particle size below 30 microns.

**Table 1:** Effect of melanging time and temperature on particle size.

Temperature (°C)	Time (h)	Particle size (microns)
16	0	50 +
	8	20–25, none below 15
	16	15–20, none below 10
	24	10–15, none below 5
24	0	50 +
	8	20–25, none below 15
	16	15–20, none below 10
	24	10–15, none below 5
38	0	50 +
	8	20–25, none below 15
	16	15–20, none below 10
	24	10–15, none below 5

### Volatile metabolite profiles from different melanging time points

**Figure 1:**
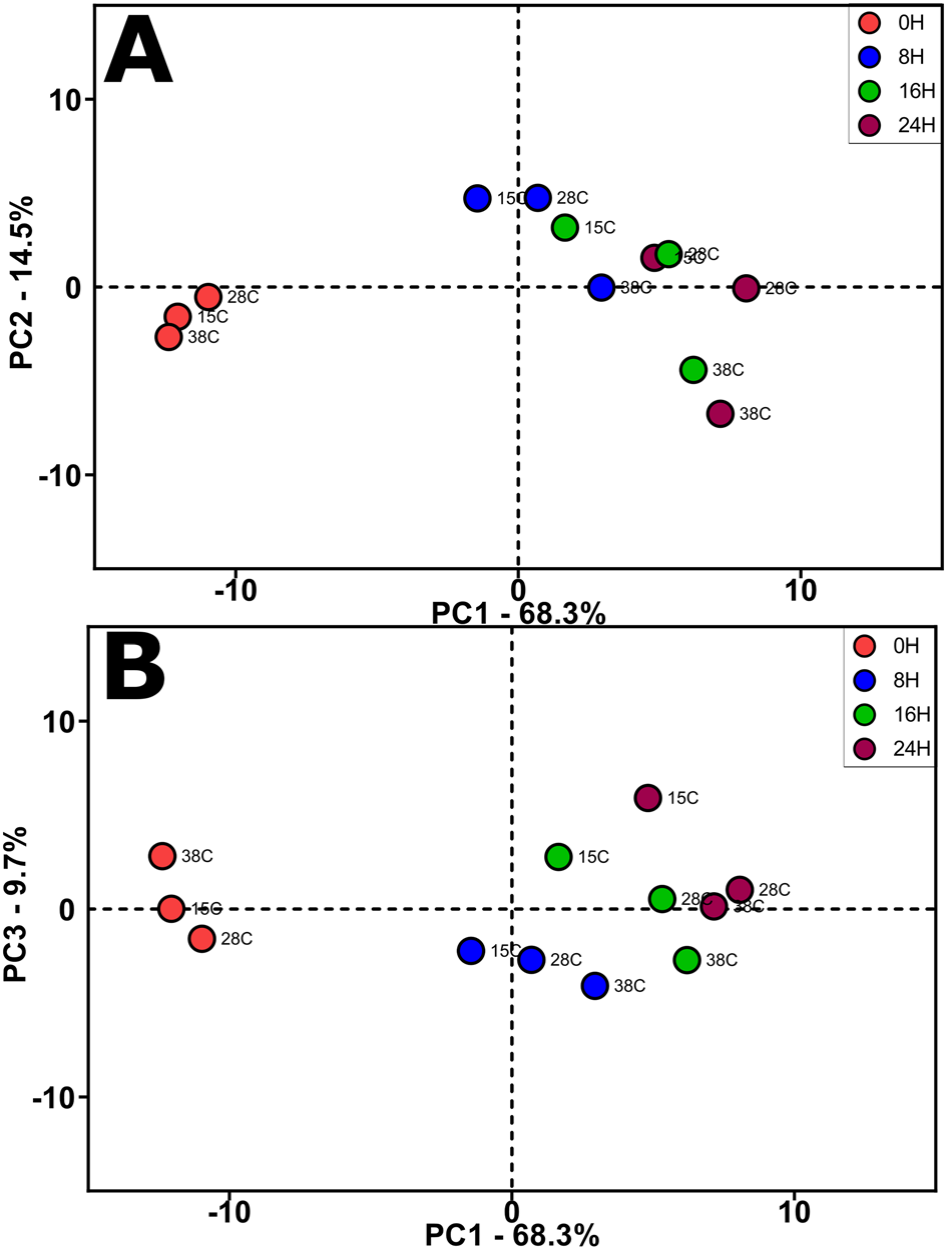
Volatile metabolite variation among the times and temperatures of chocolate during melanging. Principal component analysis (PCA) scores plots for the 88 annotated metabolites. **(A)** Top panel is PC1 vs. PC2 and **(B)** PC1 vs. PC3. Circles represent averaged volatile metabolite profiles for n = 3 HS/SPME–GC–MS extraction replicates for each chocolate time and temperature point.

To explore if the samples varied in their volatile profiles, and if this variation was time- or temperature-dependent, an untargeted metabolomics experiment was performed on the 36 samples (4 time points, 3 temperatures, 3 technical replicates) using HS/SPME GC–MS as described in the previous section. The mass spectral detection resulted in the classification of 88 chemical compounds, which were subsequently annotated using spectral matching^25^, then classified from ontological information contained in publicly available chemical databases (Supplementary Table 1). Analysis of variance (ANOVA) on the 88 compounds characterized 86/88 volatile metabolites (98%) that varied among the samples for time, 59/88 volatile metabolites (67%) that varied for temperature, and 53/88 volatile metabolites which varied for the time/temperature interaction (FDR adjusted *p* < 0.05, Supplementary Table 1). Principal Component Analysis (PCA) conducted on the 88 volatile metabolites showed that variation was attributed to time and resulted in three principal components (Fig. 1). PC1 (68.3% of the variation) was attributed to a distinct volatile profile of the samples at 0 h. PC2 (14.5%) was associated with differences at 8 and 16 h (*p* < 0.05), and PC3 (9.7%) demonstrated separation between 24 h and the other time points (*p* < 0.05).

### Chocolate volatiles’ variation due to melanging time

**Figure 2:**
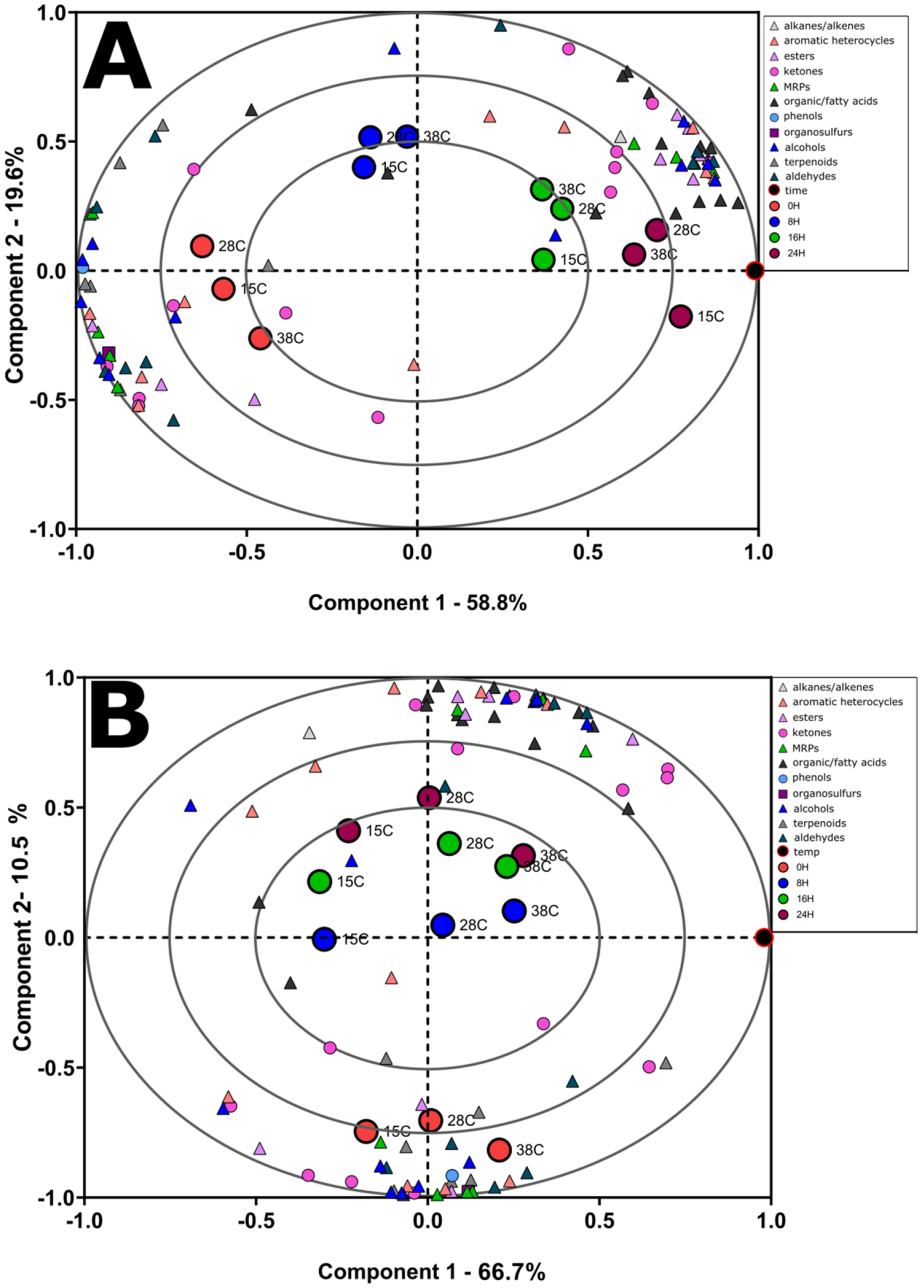
OPLS analysis of time and temperature trends among the samples. The association between time, temperature, and volatile metabolites in the chocolate was evaluated with orthogonal partial least squares (OPLS) and performed on the 88 annotated metabolites. **(A)** Biplot for the OPLS analysis based on time. Red circles denote 0-h time point, blue circles denote 8-h time point, green circles denote 16-h time point, brown circles denote 24-h time point, volatile compounds colored by known chemotype descriptors, based on temperature. **(B)** Biplot for the OPLS analysis based on time. Red circles denote 0-h time point, blue circles denote 8-h time point, green circles denote 16-h time point, brown circles denote 24-h time point, volatile compounds colored by known chemotype descriptors, based on time.

To investigate relationships between the times and temperatures of the samples and each of the 85 volatile metabolites, an orthogonal partial least squares (OPLS) model was developed with the volatile metabolite data^32^ The OPLS algorithm for time resulted in one predictive and two orthogonal components which explained 91.2% of the variation with a predictive power of Q^2^ = 96% which supported the model was not over-fit. For temperature, OPLS analysis resulted in one predictive and four orthogonal components which explained 96.9% of the variation with a predictive power of Q^2^ = 59.2% to support that the model was not over-fit, but that temperature was not predictable. Analysis of the OPLS scores and loadings for time indicate that time was linked to specific classes of volatile metabolites within the first two OPLS components, as indicated by correlation values of greater than |0.50|. The SIMCA ‘distance to model’ function was applied to characterize the metabolites with the largest contribution to explaining the variation in time (Fig. 2)^33^. The data indicated associations with pyrazines, aldehydes, terpenes, alcohols, esters, and ketones, all of which are known classes of aroma compounds important to this system.

### Trends among chemical classes

**Figure 3:**
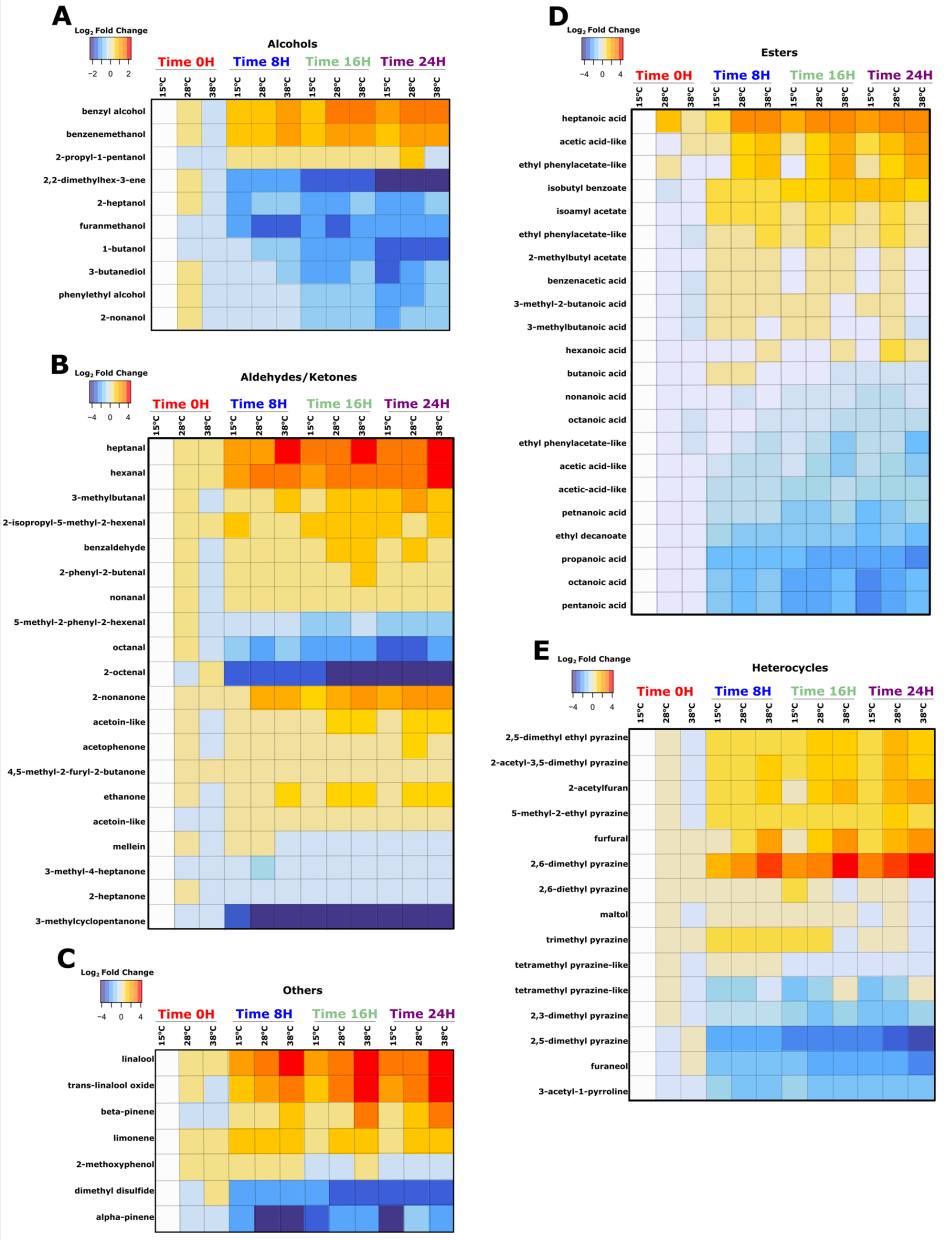
Log_2_ Fold Change analysis of metabolites related to time and temperature. Heat map of Log_2_ Fold Change value behavior of metabolites by chemical class. **(A)** Alcohols heat map of fold change values for metabolites and metabolite chemotyping (class) data. **(B)** Aldehydes/ketones heat map of fold change values for metabolites and metabolite chemotyping (class) data. **(C)** Terpenoids (and others) heat map of fold change values for metabolites and metabolite chemotyping (class) data. **(D)** Esters (organic acids and fatty acids) heat map of fold change values for metabolites and metabolite chemotyping (class) data. **(E)** Organoheterocycles heat map of fold change values for metabolites and metabolite chemotyping (class) data.

As flavor traits may be influenced by volatile chemistry, there were data taken and analyzed from four time points to evaluate the samples: 0-h, 8-h, 16-h, and 24-h. Data were evaluated to determine if trends of metabolite classes could distinguish the profiles of the chocolate masses at each time point, specifically, for lipids (to include fatty acid ester formation), Maillard reaction products (MRPs), organic acids (and their esters, which are known to be formed during thermal processing of chocolate^34^), aldehydes, ketones, and terpenes. Metabolite abundances were shown as a Log_2_ Fold Change to express the data as a profile within a sample. Therefore, a range in color denotes range in variation of a compound class within a sample, represented by very red (high) or very blue (low) (Fig. 3).

Heatmaps organized by chemical class showed that most carboxylic acids increased in abundance. At 0 h, of the 21 carboxylic acids found only 4 of them are high in abundance, but by 24 h 15 are high in abundance. Of the 22 aldehydes and ketones analyzed, 7 increase in abundance, 12 decrease, and 3 remain relatively unchanged or undergo multiple shifts in abundance. For the four terpenes found, only one (trans-linalool) increases in relative abundance by 24 h. In the case of the 10 alcohols, 5 decrease in abundance, 3 increase, and 2 remain mostly unchanged by 24 h (Fig. 3).

## Discussion

This study characterized chemical variation in chocolate mass samples that was attributed to differences in time and temperature. Specifically, the samples (i) displayed a majority particle size below 25 microns at all time points beyond 8 h, (ii) contained volatile profiles affected by melanging time and temperature, and (iii) associations between volatile metabolites and time/temperature could be determined. It is hypothesized that flavor chemistry will be principally temperature-dependent, and that data from these samples (made in a melanger) will show patterns similar to data collected on chocolate made in a refiner/conch system. Previous research has shown that flavor outcomes in refining/conching systems are strongly temperature dependent^15,35^ and have laid out a list of outcomes that constitute successful conching. These include a reduction in particle size to between 4 and 25 microns ^6,17,36^, a decrease in overall number and concentration of volatile metabolites^37^ and in an evolution/devolution of aroma compounds that can be estimated by examining the behavior of certain key indicator compounds, namely tetramethyl pyrazine, benzaldehyde, 2-methylpropanal, 2-methylheptadecane, and acetic acid (or carboxylic acids generally)^12^.

### Contribution plots at each time point

**Figure 4:**
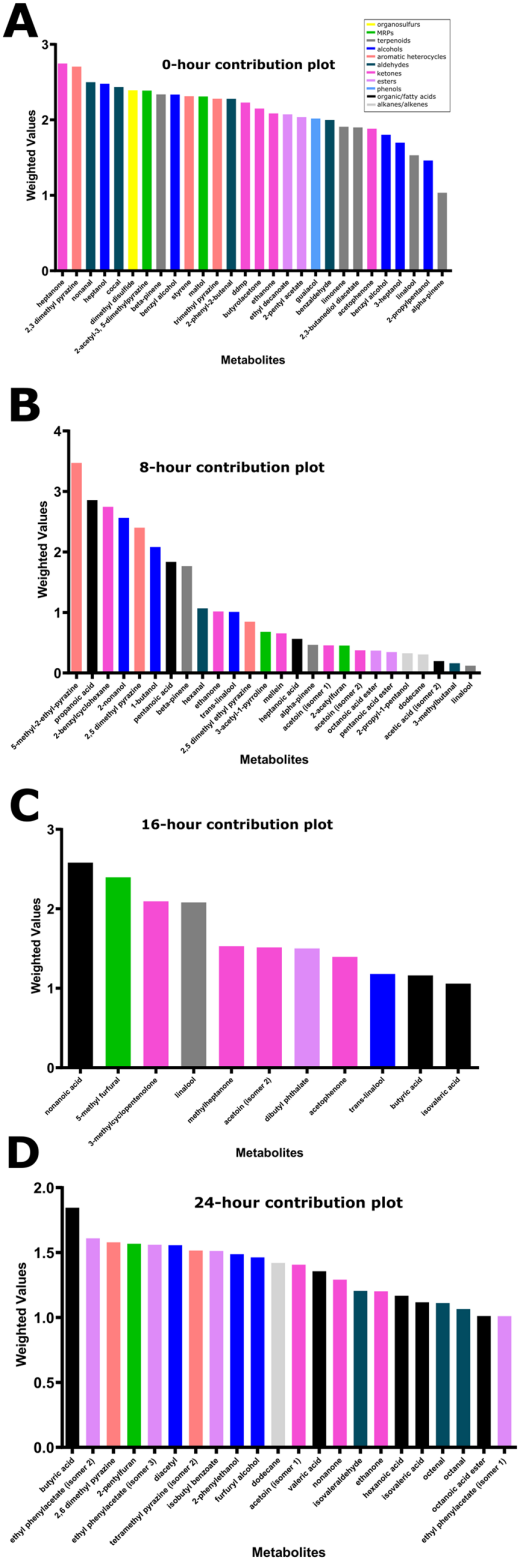
Contribution plots for each time point. The contribution scores plots display the most highly contributive metabolites for each time point, indicating deviation from the reference point. These plots show why a point in the Hotelling’s T2 plot deviate from the average. The plot shows the weighted differences between the data of the point (scaled and centered) and the average of the model. The weights are by default derived from the model. **(A)** 0-h time point**. (B)** 8-h time point. **(C)** 16-h time point. **(D)** 24-h time point.

Data were analyzed for major volatile metabolite contributors at each time point (independent from the model of the larger system, Fig. 4). At 8 h, 2-methyl-5-ethyl pyrazine and propanoic acid stand out as the two major contributors. However, what is most interesting about the 8-h sample is that many compounds in a variety of categories act as important contributors (more than one, two, or even three standard deviations). Although these compounds represent a number of chemical groups, compounds with floral and fruity flavor associations were a dominant trend at this time point, as in 2-nonanol, propanoic acid, 1-butanol, 2-acetyl furan, methyl octanoate, and linalool (Fig. 4B). At 16 h, fewer of these floral and fruity compounds were relevant, and a greater number of compounds with buttery and dairy notes, such as acetoin and acetophenone, became important contributors. The contributors at 16 h also included several carboxylic acids associated with rancid, sweaty, and unclean flavors. (Fig. 4C). The 24-h samples showed potential for a more well-rounded flavor profile, based on volatile metabolite associations with flavor literature, with multiple contributions each from compounds previously identified in literature as having a floral aromas (ethyl phenyl acetate, 2-phenylethanol, octanal), fruity aromas (2-pentylfuran, isobutyl benzoate), roasted or caramel-like aromas (2,6-dimethyl pyrazine, furfuryl alcohol, nonanone), dairy-like aromas (diacetyl, acetoin), and rancid-smelling aromas (butyric acid, valeric acid, isovaleric acid, hexanoic acid) (Fig. 4C, Supplementary Table 1). Another notable characteristic of the 24-h samples is the relative absence or low importance of pyrazines; only one pyrazine appears on the contribution plot at 24 h (Fig. 4D).

What is perhaps most interesting about the emergence and devolution of compounds is that categories of compounds do not all follow consistent patterns. Few pyrazines appear even on the 8-h contribution plot. Several carboxylic acids appear (more than on the contribution plots for the other time points) although they are different carboxylic acids than those that appear in later contribution plots. Aldehydes, esters, and ketones play a role on the 8-h contribution plot as well. Fewer compounds overall are contributors at 16 h. Ketones and carboxylic acids play a major role at this time point, although they are not the same ketones and carboxylic acids that were contributors at 8 h. Pyrazines tended to be high on the contribution plot for 24 h although only two of them appear. Esters and alcohols also appear high on the plot with contribution scores, while ketones and aldehydes have slightly lower scores and appear lower on the plot. No terpenes appear on the 24-h contribution plot.

#### Specific compounds of interest

**Figure 5:**
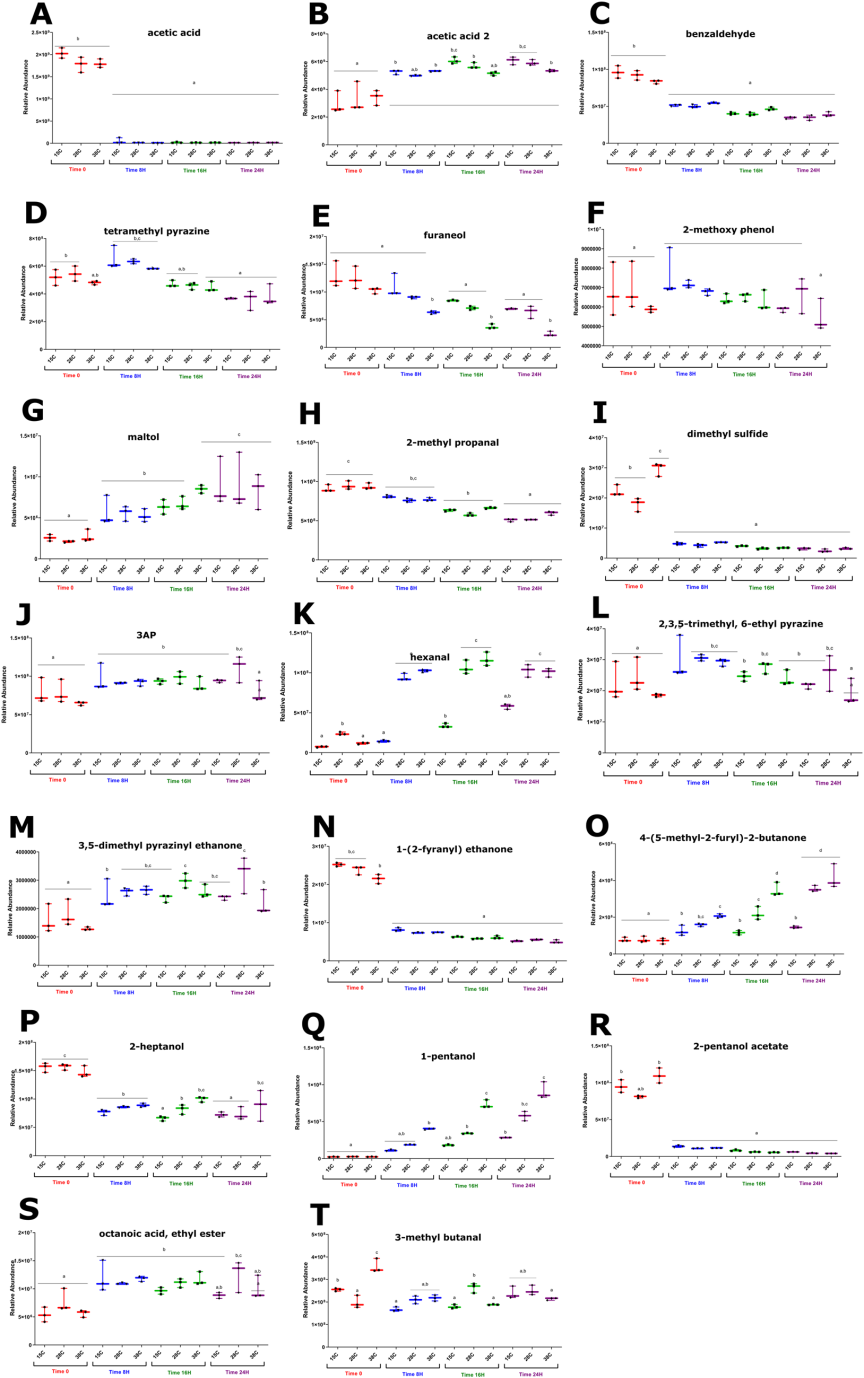
Box plots showing example metabolite variation in the system. Data are presented as the mean metabolite abundance ± standard error of the mean (S.E.M.) for n = 3 extraction replicates per chocolate sample. ANOVA with a Tukey post-hoc (*p* ≤ 0.05) was performed on the data in the box plots, and differences are denoted with letters (e.g. **A**, **B**, **C**).

Albak and Tekin^12^ asserted that a decrease in tetramethyl pyrazine, benzaldehyde, and carboxylic acids and an increase in 2-methylpropanol and 2-methyl heptadecane are signs of successful conching. The data shown here indicate that tetramethyl pyrazine levels decreased (Fig. 5D). Tetramethyl pyrazine could be viewed as a stand-in for pyrazines generally; since most pyrazines as a group followed a similar pattern of behavior, an observation of any nitrogen heterocycle should give an approximation of success. Benzaldehyde has long been recognized as an indicator of conching quality^38^; further tests will show if it serves as an indicator of melanging quality as well, but there seem to be many parallels between flavor development patterns in the two systems. In this system, its relative abundance decreases, in accordance with Albak and Tekin’s definition of successful conching (Fig. 5C).

The other indicator compounds they list not behave as Albak and Tekin predicted. The Strecker aldehyde 2-methyl propanal decreased over time (Fig. 5H); however, Counet et al.^39^ had previously shown that 2-methylpropanal does not always increase during a successful conche; it may drop slightly in actual or relative abundance. Albak and Tekin’s assertion that carboxylic acid levels should drop over time was true of some acids but not others in this system. This claim, too, has been disputed by other authors. Hoskin found that volatile fatty acid concentrations could not be predictably associated with conching time but only with rotation speed. They also showed that trained sensory panels could not detect a difference between chocolate samples with measurably distinct levels of acetic acid^40^. While these data do not align perfectly with Albak and Tekin’s work on variation of aroma content during conching^12^, a look at these compounds’ behavior in view of previous work shows nothing unexpected.

Several volatile compounds were annotated that are worth mentioning because of their relevance in previous work. Of particular interest is dimethyl sulfide, which started quite high at zero hours but then disappeared nearly completely at all temperature points by 8 h (Fig. 5I). Its disappearance is of note because it has been speculated (although not tested) that compounds of this class may be formed during conching^41^; these data indicated the opposite. The smoky off-flavor 2-methoxyphenol changed very little in relative abundance over the time measurements at all three temperatures; indeed, all three of its p-values were very high compared to those of other compounds (Supplementary Table 1, Fig. 5F). Previous work has demonstrated that this compound is conferred in the roasting stage^42^ but it can also be affected by storage conditions^43^. These data showed that an attempt to minimize this compound in the refining stage will be futile; its temperature and time dependency is simply too low. These data also affirmed previous authors’ assertions that pyrroles, such as the 3-acetyl-1H-pyrroline characteristic of dark chocolate, increased during refining and aeration^44^. This compound started low in relative abundance and climbed throughout time point measurements to moderate or high (at 28 °C) levels by 24 h (Fig. 5J).

Furaneol, shown in other works to be generated at high conching temperatures^39,41^, was present with one of the higher coefficients on the 0-h coefficient plot and had achieved an even higher placement on the regression by 24 h (Fig. 4) . Previous authors have indicated that this essential flavor compound evolves during the conching step^39,45^ while others claim it is produced during roasting^42^. These data support the latter view, as the compound appeared to be present in the system from the first measurements (Fig. 5E).

Maltol, which did not appear on any of the contribution plots, is nevertheless a compound of interest. Data showed that it increased in relative abundance over time (Fig. 5G). It has been associated with nutty, caramel flavors and has been strongly correlated in sensory studies with the perception of chocolate aroma^44^.

### Trends by chemical class

If compounds are examined by chemical class, some trends appear. Relative abundance of the less volatile carboxylic acids was generally low at time zero but had climbed higher by 24 h. It is possible that some aldehydes were oxidizing into their corresponding acids to cause this rise in relative abundance, but abundance of many aldehydes was also increasing so if this action occurred in the systems it must have been limited (Fig. 3). Those acids with greater volatility tended to decrease over the time of the experiment, but interestingly they were also generally present in greater abundance at time zero. Both detected isomers of acetic acid were very high at time zero, and while one isomer had disappeared completely by the 8-h sample, the other isomer remained in surprising abundance throughout the experiment (Fig. 5A,B).

Many of the aldehydes in the chocolate system showed a trend of low (in some cases nearly zero) relative abundance increasing at each successive time point up to 24 h (Fig. 5K). What makes these especially noteworthy is that they appeared to be more temperature-dependent than other annotated compounds; at higher ambient temperatures, their relative abundance increased more. Indeed, their p-values for the temperature variable were all in the bottom quartile of the annotated compounds. This is true of nonanal, octenal, octanal, hexanal, and heptanal (the behavior of these compounds is represented by hexanal, shown in Fig. 5K). However, relative abundance of some other aldehydes decreased (Fig. 5C).

Terpenes followed less of a pattern than other chemical classes. Further testing would be needed to predictably model the behavior of terpenes in these systems. None of the terpenes appeared to be temperature-dependent, despite their volatility.

Pyrazines displayed the strongest pattern of any annotated chemical class. As a rule, these compounds started with moderate to high abundance at 0 h and dropped off to extremely low levels by 24 h. The only exception is 2,3,5-trimethyl 6-ethyl pyrazine, which demonstrated a relatively unchanged abundance over time measurements, with slightly higher abundance at 8 h at all temperature points (Fig. 5L).

Ketones displayed both increases and decreases in relative abundance. As was the case with other isomer groups, (i.e. acetic acid, linalool) the three isomers of ethanone seemed to behave independently of one another. The (3,5-dimethylpyrazinyl) ethanone started low and climbed to moderate, while 1-(2-fyranyl) ethanone began very high but dropped to moderate by 8 h and remained there. The third isomer of ethenone, 4-(5-methyl-2-furyl)-2-butanone, was high at 0 h and climbed even higher by 24 h, especially in higher-temperature systems. (Fig. 5M,N,O).

Alcohols were also a divided class. Levels of 2-furanmethanol, 2-heptanol, and 1-pentanol all decreased in relative abundance, while all other alcohols increased in abundance or remained stable (Fig. 5P,Q). This may warrant further investigation, as previous work has shown alcohols to decrease dramatically in abundance during conching^12^. It may be that one measurable difference between conching and melanging is the increased retention of alcohols during melanging due to lower temperatures achieved during the process.

Esters as a class did not all behave identically, but the behavior trends were determined by the acetate or non-acetate character of the ester. Perhaps unsurprisingly, all the acetate esters decreased in relative abundance while the less volatile esters show the opposite trend (Fig. 5R,S).

### Possible mechanisms

Previous work on conching has indicated that if melanging affects flavor in a way similar to conching, one would expect pyrazines, pyrroles, Strecker aldehydes, and acids to decrease^12,44^ while acid, alcohol, and hydrocarbon content is variable and may depend on the volatility of the specific compounds involved^12,46^. It would also be reasonable to expect a decrease in overall number and concentrations of aroma compounds^12,37,46^. Indeed, the melanging data showed similar outcomes. Over progressive time points, (independently of temperature) nitrogen heterocycles became less relevant [those that remain at the 24-h time point are the less volatile polysubstituted rings (Fig. 5N)] and Strecker aldehydes devolved into their decomposition products [although some other aldehydes increase] (Fig. 5T)]. Volatile terpenes and alcohols disappear or have smaller contribution scores in the model (Fig. 4,5P,Q).

Many of the compounds annotated in this study fall into the category of Maillard precursors or products. As such, it is worth examining the role of Maillard reactions during melanging. Although the relative abundance of some notable Maillard products, like nitrogen heterocycles, decreased in all three temperature systems, it is theoretically possible that there were peaks in relative abundance between measured time points, or that Maillard products formed and degraded between measurements. However, it is unlikely that Maillard browning would occur in the melanging step. Early work by Rohan and later by Mohr showed that reducing sugars were the limiting factor in Maillard reaction progress in a cacao system; at the end of a typical cacao roast, reducing sugars had been entirely consumed while many free amino acids remained^47^. Although sugar is added to the melanging stage, it is typically in the form of sucrose, which is not a reducing sugar and would not contribute to Maillard reactions. Furthermore, even if sugar were added to the melanger in the form of glucose or fructose (or lactose, if milk were used), Maillard reactions would progress only sparingly. At pH values greater than 3, heat would be required to form Maillard products within the time frames described here^41^. Hoskin and Dimick^48^ previously confirmed that even traditional conching also does not reach the temperatures required for Maillard reactions (therefore melanging as performed here, which occurs at lower temperatures, also would not). This was an indication that all the Maillard compounds in evidence here were likely formed during the roasting stage and had already achieved their highest relative abundance at the beginning of the experiment. However, caramelization reactions can occur during chocolate refining, resulting in flavor compounds with similar properties to those of Maillard products^41^.

Two classes of compounds that appeared to evolve during the melanging time were aldehydes and fusel alcohols (Fig. 5Q,T). It is not surprising to see evidence of an increase in aldehyde formation, as aldehydes can be formed through the oxidation of primary and secondary alcohols, and melanging introduces great amounts of oxygen into the chocolate system. The few aldehydes that seem to be disappearing could conceivably be condensing into Schiff bases, as this reaction happens easily at a pH similar to that of chocolate mass^49^ and the dehydration component of reactions like this would contribute to some of the viscosity changes that happen during chocolate refining. The behavior of the alcohols is more difficult to explain. They are unlikely to arise from ester, aldehyde, or ketone reduction because there is no reducing agent of sufficient strength present in the system. It is probable that those alcohols which increased in relative abundance did not actually grow in amount, but rather were stable enough to be non-volatile and so their abundance increased only relative to compounds that were volatilized.

It is worth noting that in sensory analysis scenarios, compounds may act to reinforce one another or to cancel out one another’s effect in ways that may be difficult to predict from metabolomics data alone. For example, it has been established that 3-methyl butanal (Fig. 5T) and dimethyl disulfide (which appear in contribution plots for 8 and 16 h, respectively) interact to form a robustly chocolatey aroma essential for dark chocolate, although neither compound is able to produce this aroma on its own^39^. Also, some of the major contributing compounds may be below threshold levels of detectability, despite having large contribution scores. It is also possible that some compounds may play an outsized role in sensory perception because they are present far above their terminal threshold. For instance, the only two pyrazines that appear on the 24-h contribution plot are tetramethyl pyrazine (perception threshold 10 ppm in water)^39^ and 2,6-dimethyl pyrazine (perception threshold as low as 1.6 ppm in water)^50^. Without more information, it is impossible to know how these compounds might be perceived in a fat suspension and in interaction with each other or other compounds. Pyrazines in general have very low flavor thresholds^50^; it is possible that a pyrazine at even extremely low abundance may act to support other flavors even if it is not directly perceived itself. Finally, a compound that is described as having a certain flavor or aroma in isolation does not always contribute that same flavor in a system. For example, some of the same sweaty or rancid-smelling carboxylic acids that were described in the 16- and 24-h contribution plots have been identified in roasted coffee^51^ and honey^52^. However, neither of these products are traditionally associated with sweaty or rancid flavors.

## Conclusions

Several conclusions can be drawn from these data. It was determined first, that melanging is a viable method to carry out successful chocolate refining; data show that both tools result in very similar flavor outcomes when used to refine chocolate. A melanger not only sufficiently reduces the particle size of the chocolate mass but also meets similar requirements specified by previous authors for successful refining from a flavor perspective. Second, the final flavor of chocolate made in a melanger is far more dependent on time spent in the melanger than on the temperature of the chocolate system. In addition, literature previously published on conching may be successfully extrapolated to melanging. Finally, classes of chemical compounds did not behave according to unified patterns of behavior in melanging systems (with the exception of nitrogen heterocycles, which decreased in relative abundance nearly universally). Rather, within each class of compounds, behavior tended to show different trends for the more volatile compounds (often decreasing in relative abundance) than for the less volatile and poly-substituted compounds (often remaining stable or increasing in relative abundance). Subsequent work on this topic should pair targeted metabolomics analysis with sensory studies, perhaps in an effort to investigate the role of some of the less predictable classes of compounds discussed here.

Sensory work should also be performed to confirm the predictions of flavor models based on the melanging profiles presented in this work. From the data presented here, one might predict a flavor progression in the samples from time point to time point. Chocolate pulled from the melanger at 8 h would be dominated by floral and fruity notes. Allowing melanging time to continue up to 16 h would bring out a more roasted, toasty, and nutty flavor profile with rancid or sweaty undertones. A melanging time of 24 h would offer a well-rounded profile with compounds from a number of different chemical and flavor classes represented.

However, further testing is needed to confirm this prediction. Sensory tests may illuminate interactions between compounds that cannot be predicted by metabolomics data alone.

An additional recommendation that follows from this study is to determine the scalability of the conclusions drawn from these data. While these samples were taken from chocolate made on a laboratory-scale test melanger, the larger volumes of chocolate made in full-scale melangers would require correspondingly longer time points to achieve the desired results. Further testing is necessary to determine if the results shown here have a linear relationship with the volume of chocolate mass in the melanger drum, or if any mathematical relationship can be obtained.

In short, these data have illuminated the relationship between conching and melanging and shown that work previously carried out on conching can be used to inform melanging profiles. This test has been the initial investigation into the flavor impact of melanging systems. Further research is needed to elucidate the specific role of individual compounds and to manipulate variables that were not tested here.

## Supplementary information

Supplementary file1

## Data Availability

The datasets generated during and/or analyzed during the current study are available from the corresponding author on reasonable request.

## Abbreviations

PCA: Principal component analysis
ANOVA: Analysis of variance
O2PLS: Orthogonal partial least squares
HS/SPME–GC–MS: Headspace solid-phase microextraction gas chromatography mass spectrometry
