# Supplementary material for: Effects of time and temperature during melanging on the volatile profile of dark chocolate
Source: Sci Rep. 2020 Sep 10;10:14922. doi: 10.1038/s41598-020-71822-0 (PMC7483446; doi:10.1038/s41598-020-71822-0)
Supplement: Supplementary file 1 — Supplementary file1 [file 41598_2020_71822_MOESM1_ESM.pdf]

TITLE: Effects of Time and Temperature During Melanging on the Volatile Profile of Dark Chocolate

AUTHORS

Caitlin Clark[1 *], Harmonie M Bettenhausen[2], Linxing Yao[3], Adam L Heuberger[2], Martha Stone[1], Jeffrey Miller[1]

AFFILIATIONS

1: Department of Food Science and Human Nutrition, Colorado State University, Fort Collins, CO, USA; Nuance Chocolate, Fort Collins, CO

2. Department of Horticulture and Landscape Architecture, Colorado State University, Fort Collins, CO, USA

3: Proteomics and Metabolomics Facility, Colorado State University, Fort Collins, CO, USA

Supplementary Table 1: Annotated Compounds

#	Chemical Class	Compound	Common Name	Temp p-val	Time p-value	Temp*Time p-val	Aroma/Flavor from literature	Database **
6	**Alcohols (1°)**	1-Pentanol		6.51E-05	7.23E-27	2.18E-06	Oil, balsamic, vanilla, fusel, sweet, balsam	a
13		2-Furanmethanol		1.29E-05	7.32E-18	1.76E-05	Caramel, warm, cauliflower, vegetable, oily	a
14		2-Heptanol		1.76E-02	2.65E-13	7.76E-02	Leafy, coconut, herbal, peony, strawberry, musty, sweet, woody, violet, green	a
17		2-Nonanol		4.47E-01	1.55E-03	6.74E-01	Fatty, dusty, rose, fat, floral, green, clean, wet, orange, fresh, bitter, oily	a
21		2-Propyl-1-pentanol		1.85E-15	6.09E-18	4.21E-10	Found in feces	b
42		Benzenemethanol, .alpha.-methyl-, (R)-	Phenylethanol	1.88E-01	5.69E-04	2.58E-01	Gardenia, fresh, sweet, hyacinth	a
44		Benzyl alcohol		9.17E-16	1.44E-12	2.80E-12	Berry, balsamic, rose, floral, walnut, sweet, cherry, phenolic, flower, grapefruit	a
76		Phenylethyl Alcohol		1.40E-01	3.21E-05	3.69E-01	Lilac, rose, rosewater, honey, floral, spice, bitter, dried rose	a
8	**Alcohols (2°)**	2,3-Butanediol	Diacetyl	6.57E-15	3.47E-12	2.06E-09	Onion, fruit, creamy, buttery	a
24		3-Hexene, 2,2-dimethyl-		1.07E-09	1.48E-26	4.95E-03	Herbal, powerful, bitter, herb	a
16	**Aldehydes**	2-Isopropyl-5-methylhex-2-enal	Isodihydroavandulyl aldehyde	8.31E-04	6.85E-07	4.77E-01	Mint, fresh, peppermint, sweet, cool, minty, green	a

#	Chemical Class	Compound	Common Name	Temp p-val	Time p-value	Temp*Time p-val	Aroma/Flavor from literature	Database **
19		2-Octenal, (E)-		1.44E-14	1.34E-16	6.74E-09	Nut, fat, herbal, fresh, green, fatty, banana, waxy, leafy, cucumber	a
28		5-Methyl-2-phenyl-2-hexenal	Cocal	1.27E-05	9.59E-10	3.38E-03	Chocolate, bitter, butyric, cocoa, sweet, fruity, aldehydic, nut skin, green	a
36		Benzaldehyde		4.93E-01	3.67E-19	3.22E-03	Cherry, almond, sweet, burnt sugar, sharp, strong, bitter	a
37		Benzeneacetaldehyde		2.10E-03	3.30E-10	1.82E-04	Cortex, radish, naricissus, sweet, beany, honey, cocoa, nutty	a
38		Benzeneacetaldehyde, .alpha.-(2-methylpropylidene)-		4.48E-04	6.04E-08	1.59E-02	Cortex, radish, naricissus, sweet, beany, honey, cocoa, nutty	a
39		Benzeneacetaldehyde, .alpha.-ethylidene-		2.60E-02	1.29E-06	6.96E-02	Cortex, radish, naricissus, sweet, beany, honey, cocoa, nutty	a
45		Butanal, 3-methyl-		1.04E-03	3.14E-07	1.48E-08	Peach, sour, chocolate, ethereal, malt, fatty, aldehydic	a
60		Heptanal		2.55E-26	4.11E-22	5.74E-20	Citrus, ozone, fat, herbal, fresh, wine-lee, rancid, fatty, aldehydic, green	a
62		Hexanal		5.77E-19	7.82E-20	8.03E-12	Leafy, grass, sweaty, tallow, fat, fresh, fruity, aldehydic, green	a
67		Nonanal		1.26E-13	1.78E-15	5.03E-09	Citrus, lime, orange peel, rose, fat, green, fishy, waxy, fresh, orris, grapefruit	a
69		Octanal		3.15E-09	2.25E-06	2.67E-05	Lemon, citrus, soap, orange peel, fat, waxy, fatty, aldehydic, green	a
12	**Carboxylic acids**	2-Butenoic acid, 3-methyl-	Crotonic acid	2.62E-12	5.20E-14	2.43E-03	Sour, sweat, acid, stinky, sweaty, animal, rancid, tropical, feet, cheese	a
30		Acetic acid (Isomer 1)		4.33E-02	8.87E-26	4.35E-02	Sour, pungent, sharp, vinegar	a
32		Acetic acid (Isomer 2)		3.65E-01	1.32E-10	1.68E-01	Sour, pungent, sharp, vinegar	a
40		Benzeneacetic acid		3.04E-01	8.55E-07	1.69E-01	Honeysuckle, sour, waxy, floral, sweet, honey, civet, flower	a
46		Butanoic acid		9.18E-06	1.36E-04	3.38E-03	Butter, sweat, acetic, sharp, rancid, fruit, cheese	a
47		Butanoic acid, 3-methyl-		2.86E-05	3.92E-14	1.73E-05	Sour, sweat, acid, stinky, sweaty, animal, rancid, tropical, feet, cheese	a
61		Heptanoic acid		3.28E-08	2.52E-11	3.78E-04	Sour, cheesy, sweat, rancid	a
63		Hexanoic acid		5.16E-06	4.65E-13	2.85E-03	Fatty, sour, sweat, cheese	a

#	Chemical Class	Compound	Common Name	Temp p-val	Time p-value	Temp*Time p-val	Aroma/Flavor from literature	Database **
68		Nonanoic acid		3.11E-01	2.30E-03	7.67E-01	Cultured dairy, fat, waxy, green, dirty, cheese	a
70		Octanoic acid		3.55E-01	1.15E-04	8.31E-01	Cheesy, sweat, vegetable, waxy, fatty, rancid, oily	a
72		Pentanoic acid		4.63E-14	2.98E-16	9.75E-08	Sweat, putrid, sweaty, acidic, animal, rancid, sickening	a
73		Pentanoic acid, 4-methyl-		6.68E-07	1.18E-07	3.73E-04	Cheesy, sweaty, acidic, sharp, animal, fruity, green	a
77		Propanoic acid, 2-methyl-		6.57E-03	1.91E-18	6.27E-03	Rancid, butter, strawberry, cheese	a
4	**Esters**	1-Butanol methyl ester		8.14E-02	2.35E-25	5.42E-03	Oil, alcoholic, burnt, whiskey, malt, banana, fusel, fruity	a
9		2,3-Butanediol, diacetate		2.47E-01	1.79E-13	2.70E-03	None	c
20		2-Pentanol acetate		1.12E-03	4.16E-24	3.82E-05	Overripe fruit, banana, sweet, peanut, juicy	a
31		Acetic acid, 2-phenylethyl ester	Phenylethyl acetate (Isomer 1)	2.31E-02	3.15E-07	9.26E-02	Bitter, rhubarb, gardenia, musty, floral, green, sweet, herbal, leafy, fruity	a
41		Benzeneacetic acid, ethyl ester	Ethyl phenylacetate	6.42E-02	2.20E-06	9.54E-02	Grapefruit, balsam, rose, anise, chocolate, honey, melon, sweet, floral, raspberry	a
43		Benzoic acid, 1-methylpropyl ester	Isobutyl benzoate	1.36E-03	2.91E-08	2.78E-02	Powdery, musty, sweet, fruity, balsam	a
49		Dibutyl phthalate		1.91E-01	1.83E-01	6.35E-01	Faint	a
52		Dodecanoic acid, ethyl ester		1.75E-07	1.32E-12	5.30E-04	Apple, brandy, waxy, grape, oily, sweet, fruity, pear	a
71		Octanoic acid, ethyl ester		8.41E-02	2.30E-06	2.25E-01	Waxy, green, sweet, orange, aldehydic, vegetal, herbal	c
75		Phenylethyl Acetate (Isomer 2)		6.42E-02	2.20E-06	9.54E-02	Bitter, rhubarb, gardenia, musty, floral, green, sweet, herbal, leafy, fruity	a
51	**Hydrocarbons**	Dodecane		6.99E-06	7.44E-07	3.21E-04	Alkane	a
5	**Ketones**	1H-2-Benzopyran-1-one, 3,4-dihydro-8-hydroxy-3-methyl-	Mellein	2.08E-04	3.76E-09	2.64E-02	None	c
10		2-Benzylcyclohexanone		2.17E-05	4.68E-10	4.00E-03	Irritant	c
11		2-Butanone, 1-(2-furanyl)-3-methyl-		1.27E-10	3.74E-13	2.67E-07	Bitter, roasted	c
15		2-Heptanone		8.63E-05	5.75E-27	2.49E-03	Coconut, Soap, Herbal, Sweet,	a

#	Chemical Class	Compound	Common Name	Temp p-val	Time p-value	Temp*Time p-val	Aroma/Flavor from literature	Database **
18		2-Nonanone		3.18E-01	6.41E-09	2.64E-02	Woody, Fruity, Spicy, Cinnamon Soap, herbal, hot milk, earthy, soapy, weedy	a
22		3,4-Dihydro-6-methyl-2H-pyran-2-one	Ethanone (Isomer 1)	3.63E-03	1.26E-06	2.33E-01	Nutty, roasted, cooked	a
25		3-Methylcyclopentane-1,2-dione	Methylcyclopentenolone	7.24E-06	6.48E-06	7.90E-01	Heated sugar, caramel, roasted, cooked, maple syrup	c
26		4-Heptanone, 3-methyl-		3.20E-04	3.21E-04	3.14E-01	Apple, banana, cheesy	c
35		Acetophenone		2.96E-17	1.06E-16	5.89E-11	Mimosa, hawthorn, sweet, acacia, almond, pungent, flower, bitter, must	a
53		Ethanone, 1-(2-furanyl)-	Ethanone (Isomer 2)	2.28E-04	4.74E-27	1.35E-04	Caramel, tobacco, balsamic, potato, coffee, cocoa, peanut, balsam, almond, beef	c
54		Ethanone, 1-(3,5-dimethylpyrazinyl)-	Ethanone (Isomer 3)	8.46E-03	4.77E-06	2.48E-01	Nutty, hazelnut, caramel, roasted, coffee, woodsmoke	a
27	**Ketones, Alcohols**	4H-Pyran-4-one, 2,3-dihydro-3,5-dihydroxy-6-methyl-	DDMP	4.94E-01	3.54E-01	3.53E-01	Sweet, bready, cookies, dried fruit	b
34		Acetoin (Isomer 2)		4.87E-13	3.70E-20	6.84E-07	Butter, cream, milky, fatty, creamy, sweet, dairy	a
33		Acetoin (Isomer 1)		1.57E-01	2.15E-21	4.48E-06	Butter, cream, milky, fatty, creamy, sweet, dairy	a
48	**Lactones**	Butyrolactone		1.87E-09	7.19E-19	1.14E-01	Caramel, oily, fatty, sweet, creamy	a
7	**MRP***	2,3,5-Trimethyl-6-ethylpyrazine		5.00E-02	1.36E-03	9.12E-01	Roasted, baked potato, cooked	c
23		3-Acetyl-1H-pyrroline	3AP	3.15E-02	3.48E-03	3.48E-01	Dry, sweet, woody, hawthorn, nutty, popcorn, phenolic	a
55		Furan, 2-acetyl		2.28E-04	4.74E-27	1.35E-04	Caramel, tobacco, balsamic, potato, coffee, cocoa, peanut, balsam, almond, beef	a
56		Furan, 2-pentyl-		1.40E-18	1.80E-16	2.41E-12	Butter, green bean, vegetable, earthy, beany, fruity, metallic, green	a
57		Furaneol	Strawberry furone	9.90E-08	3.92E-10	6.35E-01	Sweet, burnt, brown, carmellic, cotton candy	c
58		Furfural		2.92E-09	1.80E-22	3.79E-05	Sweet, brown, woody, bready, caramellic, with a slight phenolic nuance	c
59		Furfural, 5-methyl		1.29E-10	3.74E-13	2.67E-07	Carmellic, brown	c

#	Chemical Class	Compound	Common Name	Temp p-val	Time p-value	Temp*Time p-val	Aroma/Flavor from literature	Database **
66		Maltol		1.87E-09	3.28E-26	2.20E-03	Caramel, jam, bread, cotton, sweet, fruity, baked, candy	a
78		Pyrazine, 2,3-dimethyl-		2.29E-01	6.32E-25	2.42E-04	Butter, caramel, nut, peanut butter, meat, leather, coffee, cocoa, almond, walnut	a
79		Pyrazine, 2,5-dimethyl-		8.14E-01	7.31E-25	7.35E-04	Roast beef, roasted nuts, grass, medical, cocoa, woody, medicinal	a
80		Pyrazine, 2,6-diethyl-		8.86E-02	3.55E-09	1.89E-01	Nutty, vegetable, meaty, baked	a
81		Pyrazine, 2,6-dimethyl-		7.18E-01	3.69E-25	5.55E-04	Roast beef, roasted nuts, coffee, cocoa, roasted nut	a
82		Pyrazine, 2-ethyl-5-methyl-		7.75E-01	2.95E-18	3.84E-05	Bean, fruit, sweet, coffee	a
83		Pyrazine, 3-ethyl-2,5-dimethyl-		3.31E-01	5.32E-10	1.21E-01	Roast, roasted, potato, cocoa, nutty	a
84		Pyrazine, methyl-		8.48E-09	1.58E-30	5.49E-06	Chocolate, roasted, cocoa, peanut, nutty, popcorn, green	a
85		Pyrazine, tetramethyl-		3.60E-01	3.62E-09	6.61E-01	Chocolate, burnt, lard, musty, coffee, cocoa, soybean, nutty	a
86		Pyrazine, trimethyl-		9.76E-01	2.65E-17	3.55E-04	Hazelnut, roasted, powdery, potato, musty, earthy, cocoa, peanut, nutty	a
87		Styrene		8.76E-21	8.92E-14	9.83E-16	Balsamic, gasoline, floral, sweet, plastic	a
74	**Phenols**	Phenol, 2-methoxy-		9.48E-02	3.94E-02	7.98E-01	Spice, vanilla, smoky, sweet, woody, phenolic, medicinal, smoke	a
50	**Sulfur Compounds**	Disulfide, dimethyl		1.14E-06	5.26E-22	1.23E-07	Fruity, herbal, vegetal, sulfurous, fatty, floral, nutty, meaty, spicy	a
1	**Terpenes**	(3R,6S)-2,2,6-Trimethyl-6-vinyltetrahydro-2H-pyran-3-ol	Linalool (Isomer 1)				Floral, fresh, lavender	a
29		6,6-Dimethyl-2-vinylidenebicyclo[3.1.1]heptane	Beta-pinene (Isomer 2)	3.58E-04	1.04E-04	7.68E-01	Resinous, fresh, green, hay, woody, pine, dry	a
2		Alpha-Pinene		2.57E-03	5.41E-17	4.00E-01	Harsh, turpentine, minty, fresh, earthy, sweet, woody, terpene, pine, camphor	a
3		Beta-Pinene (Isomer 1)		6.55E-02	5.89E-21	5.00E-02	Resinous, resin, turpentine, green, hay, woody, pine, dry	a
64		Limonene		4.19E-06	2.08E-12	3.08E-08	Mint, lemon, citrus, orange, fresh, sweet	a
65		Linalool (Isomer 2)		1.22E-01	5.75E-10	2.48E-01	Citrus, lemon, orange, floral, sweet,	a

#	Chemical Class	Compound	Common Name	Temp p-val	Time p-value	Temp*Time p-val	Aroma/Flavor from literature	Database **
88		Trans-Linalool oxide (furanoid)		5.50E-01	9.23E-08	1.15E-01	woody, blueberry, bois de rose, lavender, green Floral, flowers	a

*MRP = Maillard Reaction Product

** = a – Flavor DB, b – HMDB, c – Good Scents Company
